# Involvement in data initiatives

**DOI:** 10.23889/ijpds.v8i2.2234

**Published:** 2023-09-14

**Authors:** Elisa Jones

**Affiliations:** 1 University of Liverpool, Liverpool, United Kingdom

Summer 2021. #NhsDataGrab is trending on Twitter and over a million people opt out of NHS data-sharing in one month in a backlash against a government plan: General Practice Data for Planning and Research. The scheme proposes that GP anonymised health data for everyone in England will be made available to researchers and companies for healthcare research and planning. The plan is now on hold, much to the dismay of data scientists who believe that data saves lives.

It has been suggested that greater public participation around the sharing of health data will help to alleviate public concerns, and may prevent more members of the public opting out. Health data initiatives have involved members of the public in different ways, from “citizen juries” to public panels and advisory boards.

Using ethnographically-informed qualitative case studies, this project takes a closer look at involvement approaches in different data sharing initiatives. The case studies include: citizen juries that asked jurors to consider different real-world data initiatives; a public panel set up by a regional databank that carries out data linking; and an advisory board of members of the public at a national data institution. I have carried out observations of the involvement activities, and conducted semi-structured 1:1 interviews with those who organise and have taken part in the activities.

I have recently completed data collection, with analysis ongoing. The analysis is thematic and primarily inductive, using principles of grounded theory and drawing on Silverman’s constant comparative method. Key themes, patterns, and variations are currently being noted and developed. Current areas of interest include: epistemic or democratic reasoning for activities; the roles that the different actors play in participation and how contributors are changed by their involvement.

